# Potential Beneficial Effects of *Sargassum* spp. in Skin Aging

**DOI:** 10.3390/md20080540

**Published:** 2022-08-22

**Authors:** Min-Kyeong Lee, Heeyeon Ryu, Ji Yun Lee, Hyeon Hak Jeong, Jiwon Baek, Ji Yun Van, Myeong-Jin Kim, Won-Kyo Jung, Bonggi Lee

**Affiliations:** 1 Department of Food Science and Nutrition, Pukyong National University, Daeyeon-dong, Nam-gu, Busan 48513, Korea; 2 Division of Biomedical Engineering and Research Center for Marine Integrated Bionics Technology, Pukyong National University, Daeyeon-dong, Nam-gu, Busan 48513, Korea

**Keywords:** brown seaweeds, sargassum, skin aging, cosmeceutical

## Abstract

Seaweeds are receiving much attention as a rich source of bioactive compounds with cosmeceutical potential. Recent studies have revealed that *Sargassum* spp., a genus of brown algae in the family Sargassaceae, has multiple functions in preventing and improving skin aging. *Sargassum* spp. contains many bioactive compounds, such as fucoidan, fucoxanthin, terpenoids, flavonoids, and meroterpenoids. These *Sargassum* spp. extracts and derivative compounds have excellent potential for skincare, as they exhibit skin health-promoting properties, including antioxidants, anti-inflammation, whitening, skin barrier repair, and moisturizing. Therefore, searching for bioactive compounds in marine resources such as *Sargassum* spp. could be an attractive approach to preventing and improving skin aging. The current review focused on the various biological abilities of *Sargassum* extracts or derived compounds for anti-skin aging.

## 1. Introduction

*Sargassum*, a genus of brown algae in the Sargassaceae family, is a seaweed commonly found in tropical and subtropical coastal areas, with more than 400 species distributed in almost all marine basins [1,2]. In particular, massive *Sargassum* biomass found from the Atlantic Ocean to the Caribbean Sea is called the Great Atlantic Sargassum Belt and is responsible for stranding numerous algae in coastal areas, causing severe environmental, ecological, and economic problems [3,4]. The massive influx of *Sargassum* and subsequent decaying algae on the beach could directly impact the affected area as it has harmful effects on the health of the local and traveling populations (respiratory, skin, and neurological diseases caused by the release of hydrogen sulfide and ammonia), tourism, and fishing activities [5,6,7]. Also, removing *Sargassum* biomass from beaches is problematic because it is expensive. Therefore, researching alternatives to increase the value of *Sargassum* biomass reaching the coasts could not only render *Sargassum* tides an economic opportunity but also solve their related environmental problems.

*Sargassum* spp. are known to have a wide range of bioactive metabolites with potential applications in the biofuel, nutraceutical, pharmaceutical, and cosmetic industries [8]. Of the more than 400 species of *Sargassum*, only 78 species were investigated for their functional and phytochemical properties, of which most studies focused on 18 species [9]. The main *Sargassum* spp. include *S. horneri*, *S. fusiforme*, *S. muticum*, *S. pallidum*, *S. siliquastrum*, *S. thunbergii*, *S. fulvellum*, *S. polycystum*, and *E. stolonifera* and have long been consumed as food or traditional medicine in many countries to prevent or treat a variety of diseases (Table 1) [9]. *Sargassum* spp. are rich in carbohydrates, proteins, amino acids, vitamins, minerals, dietary fiber, and carotenoids. Additionally, approximately 200 biologically active compounds in *Sargassum* spp. have been reported, which include but are not limited to meroterpenoids, terpenoids, flavonoids, sulfated polysaccharides, polyphenols, pheophytin, fucoidans, phlorotannins, glycolipids, and sterols. These compounds exhibit various biological activities such as anti-inflammatory, antifungal, antiviral, antibacterial, antioxidant, anti-melanogenesis, anti-metabolic syndrome, neuroprotective, and anti-bone loss effects [9,10,11,12,13,14].

Although numerous studies have investigated the beneficial effects of natural product-derived extracts or compounds on skin aging, the roles of *Sargassum* spp. in skin aging have not yet been extensively studied. Nevertheless, recent research has shown that *Sargassum* spp. play promising roles in preventing and improving skin aging. Thus, this review aims to explore the potential effects of *Sargassum* spp. on the prevention and improvement of skin aging and their underlying molecular mechanisms. We especially focused on the beneficial effects of *Sargassum* extracts or derived compounds on the prevention of skin aging including whitening, moisturization, skin barrier repair, photoprotection, antioxidants, and anti-inflammatory functions. Literature searches were limited to published papers or reports written in English, and the reference lists of all relevant research and review articles were manually searched. Relevant keywords for the terms “brown seaweeds”, “*Sargassum*”, and “*Sargassum* spp.” were analyzed in association with other terms such as “skin aging”, “antioxidant”, “anti-inflammation”, “skin barrier repair”, and “anti-melanogenesis”.

## 2. Bioactive Functions of *Sargassum* spp.

### 2.1. Antioxidant and Photoprotective Activities

Reactive oxygen species (ROS)-induced intracellular and extracellular oxidative stress promotes skin aging characterized by atypical pigmentation and wrinkles. Skin aging is commonly discussed as related to ultraviolet (UV) exposure, as UV radiation triggers photoaging of the skin by increasing oxidative stress in cells. Therefore, various in vitro and in vivo studies investigated the effects of *Sargassum* spp. extract on antioxidant and skin photoprotection (Table 2).

#### 2.1.1. *Sargassum muticum*

A study examined the photoprotective effects of the ethyl acetate fraction of *S. muticum* against UVB radiation-induced skin damage and photoaging in hairless-1 (HR-1) mice and human keratinocytes [25]. When applied to UVB-irradiated HR-1 mice, the oral injection of ethyl acetate fraction (100 mg/kg body weight) significantly improved the average length and depth of wrinkles and inhibited the increase in epidermal thickness [25]. Also, collagen bundle formation was markedly increased in UVB-irradiated HR-1 mice administrated with the ethyl acetate fraction. In the same study, ethyl acetate fraction pretreatment at least partially reversed the elevated protein level and activity of matrix metalloproteinase-1 (MMP-1) and the binding of activator protein-1 (AP-1) to the MMP-1 promoter in UVB-irradiated HaCaT keratinocytes [25]. These results suggest that the ethyl acetate fraction mediates the photoprotective effect of *S. muticum* extract.

#### 2.1.2. *Sargassum cristafolium*

The effects of *S. cristafolium* extract on UVA-induced skin damage were investigated using BALB/c mice [47]. The data showed that the treatment of *S. cristafolium* extract significantly inhibited the UVA-mediated increases in the levels of skin wrinkles and desquamation and induced the skin healing process [47]. It is assumed that the underlying mechanisms of *S. cristafolium* extract-mediated protection of skin damage involve the up-regulation of interleukin-10 (IL-10) production and down-regulation of the pro-inflammatory cytokines including tumor necrosis factor-α (TNF-α) and IL-6 expression [47]. Additionally, the minimum inhibitory concentration of *S. cristafolium* extract against *Staphylococcus aureus* was 1302 µg/mL, indicating excellent antibacterial activity [47]. Furthermore, the researchers confirmed the presence of fucoxanthin in *S. cristafolium* extract by LC-MS analysis [47]. These results suggest that *S. cristafolium* extract exerts photoprotective efficacy by modulating anti-inflammatory and pro-inflammatory cytokines. However, it is necessary to further study the photoprotective activity of purified compounds such as fucoxanthin.

#### 2.1.3. *Sargassum siliquastrum*

Low molecular weight (LMW) fucoidan with relatively short chain lengths is known as an effective antioxidant with various biological activities such as anti-inflammatory and antibacterial effects and supporting immune regulation [55,56,57]. A recent study investigated the photoprotective properties of the fucoidan fraction of *S. siliquastrum* (SSQC) against UVB-stimulated skin damage using human keratinocytes [31]. The results showed that SSQC4 had the lowest molecular weight among the fucoidan fractions of *S. siliquastrum* (SSQC1 [40–160 kDa], SSQC2 [50–95 kDa], SSQC3 [25–75 kDa], SSQC4 [8–25 kDa]) obtained by step gradient ethanol precipitation and had the best bioactive effects. SSQC4 (25–100 μg/mL) treated to UVB-exposed HaCaT keratinocytes dose-dependently reduced elevated intracellular ROS levels and inhibited the mitochondrial-mediated apoptosis pathway [31]. The photoprotective effects of SSQC4 were associated with activation of the nuclear factor erythroid 2-related factor 2 (Nrf2)/heme oxygenase-1 (HO-1) antioxidant pathway [31]. 

#### 2.1.4. *Sargassum fusiforme*

A study examined the UVB-protective effect of polysaccharide purified from *S. fusiforme* using HaCaT keratinocytes [48]. Treatment of polysaccharide (31.25–125 μg/mL) reduced intracellular ROS levels and pro-inflammatory cytokine (IL-1β, IL-6, and TNF-α) secretion in UVB-stimulated HaCaT cells [48]. In addition, polysaccharide significantly reduced the mRNA expression levels of MMP-1,3 and 9 involved in extracellular matrix degradation in UVB-stimulated HaCaT cells [48].

Another study evaluated the effects of sulfated polysaccharides from Celluclast-assisted extracts of *S. fusiforme* on UVB-stimulated photoaging using human dermal fibroblast (HDF) cells [20]. Sulfated polysaccharides (25–100 μg/mL) improved the cell viability of UVB-irradiated HDF cells by dose-dependently decreasing intracellular ROS levels [20]. Additionally, sulfated polysaccharides significantly protected collagen breakdown by reducing intracellular collagenase and elastase activities and MMPs expression levels in UVB-irradiated HDF cells [20]. The protective effects of sulfated polysaccharides on UVB-induced skin damage are associated with inhibition of the nuclear factor-κB (NF-κB), AP-1, and mitogen-activated protein kinase (MAPK) signaling pathways [20].

#### 2.1.5. *Sargassum confusum*

A study examined the UVB-protective effect of fucoidan from *S. confusum* using human keratinocytes [29]. *S. confusum* was extracted using Celluclast, and four fucoidan fractions were obtained by step gradient ethanol precipitation. The results showed that the treatment of the LMW fucoidan fraction (20 kDa) recovered the cell viability while suppressing intracellular ROS generation in UVB-irradiated HaCaT keratinocytes. Additionally, LMW fucoidan fraction inhibited UVB-stimulated sub-G1 cell content, apoptotic body formation, and DNA damage by blocking the activation of the mitochondrial-mediated apoptosis signaling pathway [29]. Furthermore, LMW fucoidan fraction protected the stratum corneum hydration damage by inhibiting the upstream mediators of the UVB-stimulated inflammatory responses in HaCaT keratinocytes [29]. These photoprotective effects of LMW fucoidan fraction could be attributed to the activation of the antioxidant enzyme Nrf2 and inhibition of the NF-κB and MAPK signaling pathways [29].

#### 2.1.6. *Sargassum coreanum*

A recent study tested the effects of fucoidan derived from *S. coreanum* on UVB-stimulated oxidative stress in HaCaT keratinocytes [49]. Fucoidan fraction (nearly 50 kDa) treatment in UVB-exposed HaCaT cells significantly inhibited the intracellular ROS levels [49]. UVB irradiation also increased apoptotic body formation and DNA damage through activation of the mitochondrial-mediated apoptosis signaling pathways, whereas the treatment of fucoidan fraction reversed these properties [49]. These beneficial effects of the fucoidan fraction are partially ascribed to activation of the Nrf2/HO-1 signaling pathway [49].

#### 2.1.7. *Sargassum horneri*

Several studies have demonstrated that *S. horneri* extracts and derived compounds are beneficial in inhibiting UVA- and UVB-induced oxidative stress and photoaging [15,50,51,52,53,54]. A study tested whether the treatment of *S. horneri* methanol extracts containing 6-hydroxy-4,4,7a-trimethyl-5,6,7,7a-tetrahydrobenzofuran-2(4H)-one (HTT) and apo-9′-fucoxanthinone affected UVB-induced photodamage in human keratinocytes [15]. The methanol extract of *S. horneri* enhanced the cell viability of human keratinocytes by blocking the intracellular ROS production induced by UVB exposure [15]. In addition, UVB irradiation increased the formation of apoptotic bodies and populations in sub-G1 hypodiploid cells and early apoptotic cells through regulation of the expression of B-cell lymphoma-2 and BCl-2 associated X, whereas treatment with *S. horneri* methanol extracts reversed these properties [15]. The anti-photoaging process that inhibits intracellular ROS generation and apoptosis in UVB-exposed HaCaT keratinocytes involves inhibition of NF-κB p65 activation partly by activating the Nrf2/HO-1 signaling pathway [15].

Another study investigated the effects of HTT isolated from *S. horneri* on UVB-mediated oxidative damage using HDF cells [50]. The data showed that HTT treatment inhibited UVB-mediated intracellular ROS generation, mitochondrial hyperpolarization, and apoptotic body formation [50]. HTT treatment in UVB-exposed HDF cells significantly reduced the expression levels of NF-κB and MAPK signaling proteins in a dose-dependent manner and also down-regulated pro-inflammatory cytokines such as IL-1β, IL-6, IL-8, IL-33, and TNF-α [50]. Additionally, HTT treatment significantly inhibited the mRNA expression levels of MMP-1, MMP-2, MMP-3, MMP-8, MMP-9, and MMP-13 in a dose-dependent manner and also down-regulated collagenase and elastase activities in UVB-exposed HDF cells [50]. Thus, the beneficial effects of HTT may be achieved by suppressing inflammatory responses and stimulating the degradation of extracellular matrix components. 

The photoprotective effects of (−)-loliode compounds isolated from *S. horneri* have been reported in both in vitro and in vivo models [51]. (−)-loliode down-regulates intracellular ROS levels, improving cell viability and inhibiting apoptosis in UVB-irradiated HaCaT keratinocytes [51]. Additionally, (−)-loliode significantly attenuated oxidative damage, stimulated collagen synthesis, and decreased MMP-1, MMP-2, MMP-8, MMP-9, and MMP-13 expressions in UVB-exposed HDF cells [51]. Furthermore, in vivo tests using zebrafish showed that (−)-loliode significantly inhibited the levels of ROS, nitric oxide (NO), lipid peroxidation, and apoptosis increased by UVB irradiation [51]. These in vitro and in vivo results suggest that (−)-loliode has strong photoprotective activity.

Fucoidans are sulfated polysaccharides found in the cell walls of brown algae and are known to exhibit various biological activities [58]. A recent study evaluated the effects of LMW fucoidan from *S. horneri* on UVB-induced oxidative stress and photoaging [52]. The LMW fucoidan treatment in UVB-exposed HaCaT keratinocytes improved cell viability by inhibiting intracellular ROS production [52]. Furthermore, the LMW fucoidan modulated mitochondrial-mediated apoptotic pathways, significantly suppressing UVB-stimulated apoptotic body formation, sub-G1 accumulation of cells, and DNA damage [52]. These beneficial effects of the LMW fucoidan are partially ascribed to enhancing intracellular antioxidant defenses by activation of the Nrf2/HO-1 signaling pathway [52].

Other previous studies investigated the roles of chromene compounds sargachromenol and sargachromanol E isolated from *S. horneri* in UVA-irradiated HDF cells [53,54]. Sargachromanol E decreased UVA-induced intracellular ROS production, membrane protein oxidation, and lipid peroxidation [54]. In addition, sargachromenol and sargachromanol E reduced the expression of MMP-1, MMP-2, and MMP-9 in UVA-irradiated HDF cells, and these reductions were associated with an increase in tissue inhibitor of metalloprotease-1 (TIMP-1) and TIMP-2 expression [53,54]. Furthermore, the transcriptional activation of AP-1, which was enhanced by UVA, was inhibited by treatment with sargachromenol and sargachromanol E [53,54]. These results suggest the potential of sargachromenol and sargachromanol E to exert a photoprotective effect, an area that may explore in future studies.

### 2.2. Anti-Inflammatory Activity

Dysregulation of inflammatory signals is associated with the pathogenesis of inflammatory skin abnormalities [59]. Inflammatory responses induced by UV exposure, a causative factor in skin aging and skin cancer, have also been shown to involve modulators of inflammatory signaling, such as Toll-like receptor 4 [60]. Therefore, various studies on the anti-inflammatory effects of *Sargassum* spp. extract were summarized (Table 3).

#### 2.2.1. *Sargassum fulvellum*

A study investigated the protective effect of the ethyl acetate fraction of ethanol extract of *S. fulvellum* on UVB-induced inflammation in human keratinocytes and BALB/c mice [38]. In HaCaT cells, the treatment of the ethyl acetate fraction (30–100 μg/mL) reduced the UVB-induced production of the pro-inflammatory proteins such as cyclooxygenase-2 (COX-2), TNF-𝛼, inducible nitric oxide synthase (iNOS), and pro-inflammatory mediators such as NO, prostaglandin E2 (PGE2) [38]. In addition, ethyl acetate fraction decreased intracellular ROS production and increased cellular defense ability against oxidative stress through the upregulation of antioxidant enzymes such as catalase and Cu/Zn-superoxide dismutase [38]. Furthermore, in UVB-induced damaged skin of BALB/c mice, treatment with ethyl acetate fraction (3–10 μg) downregulated the expression of COX-2, iNOS, TNF-𝛼, NO, and PGE2 [38]. These data indicate that ethyl acetate fraction has a protective effect against UVB-induced inflammation by modulating the expression of inflammatory cytokines and enhancing antioxidant defense systems.

Another study revealed an anti-inflammatory mechanism of the hexane fraction of *S. fulvellum* against lipopolysaccharide (LPS)-stimulated mouse-derived macrophages and phorbol 12-myristate 13-acetate (PMA)-induced mouse ear edema [61]. Hexane fraction (45 μg/ear) significantly reduced the edema of the mouse ears. In addition, treatment of hexane fraction (10–50 μg/mL) decreased the secretion of NO, iNOS, TNF-α, IL-1β, and IL-6 in LPS-treated RAW264.7 cells [61]. Further studies revealed that hexane fraction-mediated anti-inflammatory properties are achieved partly by inhibition of NF-κB activation through inactivation of the MAPK and protein kinase B (Akt) pathways [61].

#### 2.2.2. *Sargassum horneri*

A recent study evaluated the protective effects of alginate extracted from *S. horneri* (SHA) on urban aerosols (Chinese fine dust, CFD) using human keratinocytes and mouse macrophages [16]. SHA (25–100 μg/mL) significantly reduced the protein levels of COX-2, PGE2, IL-6, and TNF-α and intracellular ROS levels in HaCaT keratinocytes treated with skin inflammation-inducing CFD [16]. These anti-inflammatory effects are associated with inhibition of the NF-κB and MAPK signaling pathways [16]. In the same study, when the culture medium derived from CFD-stimulated HaCaT cells (with or without SHA treatment) was added to RAW264.7 cells, the SHA-treated culture medium inhibited inflammatory factors (PGE2, NO, iNOS, and COX-2) and pro-inflammatory cytokines (TNF-α, IL-6, and IL-1β) [16]. This suggests that SHA is effective in protecting keratinocytes from CFD-induced inflammation.

#### 2.2.3. *Myagropsis myagroides*

Studies using ICR mice and mouse-derived macrophages showed the effect of ethanolic extract from *M. myagroides* on anti-inflammatory responses [62]. Treatment of ethanolic extract from *M. myagroides* (10–100 μg/mL) reduced the levels of pro-inflammatory cytokines including IL-1β, TNF-α, and IL-6, and pro-inflammatory mediators including NO, PGE2, COX-2, and iNOS in activated RAW264.7 macrophages [62]. This phenomenon correlates with NF-κB inhibition by interfering with the Akt and MAPK pathways [62]. In addition, in mouse ears with edema, by applying PMA evenly to the inner surface of the ears, treatment of ethanolic extract from *M. myagroides* (90 μg/ear) resulted in a significant improvement in skin edema [62]. Further studies revealed that Eckol and 6,6’-bieckol are one of the major compounds in ethanolic extract from *M. myagroides*. Of these, only 6,6’-bieckol decreased the expression levels of iNOS and COX-2, and PMA-induced ear edema [62], demonstrating that 6,6′-biechol contributes to the anti-inflammatory effect of ethanolic extract from *M. myagroides*.

Another study observed the molecular mechanism of anti-inflammatory effects of the ethyl acetate fraction of *M. myagroides* in ICR mice and mouse-derived macrophages [63]. The treatment of ethyl acetate fraction (25–100 μg/mL) reduced the secretion of inflammatory mediators (PGE2, iNOS, COX-2, and NO) and pro-inflammatory cytokines (IL-1β, IL-6, and TNF-α) in LPS-treated macrophages [63]. In the same study, treatment with ethyl acetate fraction (90 μg/mL) reduced PMA-induced ear edema in mice [63]. The molecular mechanisms underlying ethyl acetate fraction-mediated anti-inflammatory effects include, but are not limited to, the inhibition of extracellular signal-regulated kinase (ERK), Akt, and NF-κB signaling [63]. Further study showed that eckol and phlorofucofuroeckol B are one of the main compounds in ethyl acetate fraction. Of these, only phlorofucofuroeckol B (12–24 μg/mL) suppressed the protein levels of iNOS and COX-2, contributing to the inhibition of ear edema [63]. Phlorofucofuroeckol B contributes to the anti-inflammatory effect of ethyl acetate fraction.

Subsequent studies investigated the anti-inflammatory mechanism of sargaquinoic acid isolated from *M. myagroides* [64]. Sargaquinoic acid (0.3–1.2 μM) down-regulated IL-1β, IL-6, TNF-α, and NO, PGE2, iNOS, and COX-2 expression in LPS-treated RAW264.7 cells [64]. The inhibitory effect of these inflammation-related factors is related to the inhibition of NF-κB mediated by the activation of the Nrf2/HO-1 signaling pathway and the inhibition of the activation of the ERK and Akt pathways [64]. 

#### 2.2.4. *Ecklonia stolonifera*

A study tested the anti-inflammatory activity of ethanol extract of *E. stolonifera* against LPS-stimulated inflammatory responses in macrophages [44]. In RAW264.7 cells stimulated by LPS, treatment with ethanol extract (50–200 μg/mL) decreased the secretion of pro-inflammatory cytokines such as TNF-α, IL-1β, and IL-6. Additionally, inflammatory mediators including COX-2, NO, PGE2, and iNOS also tended to be inhibited [44]. The protective effect of ethanol extract against LPS-induced inflammation was associated with inhibition of NF-κB, AP-1, and MAPK signaling pathways [44]. Among phlorofucofuroeckol A and B, dieckol, and eckol isolated from ethanol extract, phlorofucofuroeckol A and B significantly contributed to the anti-inflammatory activity of ethanol extract [44].

#### 2.2.5. *Sargassum macrocarpum*

A recent study investigated the anti-inflammatory activity of sargahydroquinoic acid isolated from the marine brown alga *S. macrocarpum* using in vitro and in vivo mouse models [65]. Treatment with sargahydroquinoic acid (0.4–0.8 μM) dose-dependently reduced inflammation-related factors including COX-2, NO, PGE2, iNOS, TNF-α, IL-1β, and IL-6 in LPS-activated RAW264.7 cells [65]. It was also found that sargahydroquinoic acid induced Nrf2/HO-1 activation and inhibited the Akt-mediated NF-κB pathway [65]. In addition, when the concentration of pro-inflammatory cytokines was measured after the administration of sargahydroquinoic acid (10–15 mg/mL) to mice, the blood levels of TNF-α, IL-1β, IL-6, and IL-17 were significantly reduced [65], suggesting that sargahydroquinoic acid is a strong anti-inflammatory compound found in *S. macrocarpum.*

#### 2.2.6. *Sargassum fusiforme*

A study investigated the anti-inflammatory effect of polysaccharides derived from *S. fusiforme* in LPS-induced RAW264.7 cells [24]. The results showed that polysaccharides (25–100 μg/mL) markedly inhibited NO production and enhanced cell viability in LPS-induced RAW264.7 cells [24]. Additionally, polysaccharides decreased the production of PGE2, TNF-α, IL-1β, and IL-6 and inhibited the protein expression of iNOS and COX-2 in LPS-stimulated RAW264.7 cells [24]. These results suggest that polysaccharides derived from *S. fusiforme* have potential anti-inflammatory activity.

#### 2.2.7. *Sargassum micracanthum*

A previous study investigated the anti-inflammatory effect of sargachromenol isolated from *S. micracanthum* in LPS-stimulated RAW264.7 macrophages [66]. The data showed that sargachromenol (12.5–100 μM) significantly inhibited LPS-induced production of NO and PGE2 and protein expression levels of iNOS2 and COX-2 in a dose-dependent manner [66]. In addition, the cytoplasmic loss of inhibitor kappa B alpha protein increased by LPS was inhibited by sargachromenol treatment [66], indicating that the protective effect of sargachromenol on LPS-induced inflammation is partially related to the inhibition of NF-κB signaling pathways. 

#### 2.2.8. *Sargassum binderi*

The anti-inflammatory effects of sulfated polysaccharides derived from *S. binderi* were reported in vitro and in vivo models [67]. Treatment of sulfated polysaccharides (25–100 μg/mL) decreased NO production and protein expression levels of iNOS and COX-2 in LPS-stimulated RAW264.7 cells [67]. Furthermore, in vivo tests using zebrafish larvae indicated that sulfated polysaccharides markedly inhibited cell death and NO production increased by LPS treatment [67]. Therefore, sulfated polysaccharides derived from *S. binderi* have anti-inflammatory effects through the inhibition of inflammatory mediators.

### 2.3. Moisturization and Skin Barrier Repair

Not only does the skin barrier have major functions to protect the body against physical and chemical injury, but it also prevents the loss of body water and other substances. Skin dryness results in observable changes including redness, flakes, dry white patches, a lack of luster surface, cracks, and even fissures [68]. Moisturization and skin barrier repair are considered one of the primary indicators to evaluate skin health. Recent research exhibited that various extracts from *Sargassum* showed beneficial effectsfor moisturization and skin barrier repair [19,29,69,70]. We summarized the potential ability of *Sargassum* spp. for moisturization and skin barrier repair (Table 4).

#### 2.3.1. *Sargassum muticum*

The inhibitory activities of *S. muticum* extracts against enzymes involved in the degradation of the extracellular matrix, such as elastase, collagenase, and hyaluronidase, were recently reported [28]. The dermis and epidermis of the skin are composed of collagen and elastin, which play a role in making the skin firm and supple [28]. Hyaluronic acid also plays an essential role in maintaining the hydration and plasticity of the skin [73]. The ethyl acetate fraction isolated from *S. muticum* did not affect elastase activity, whereas it strongly inhibited collagenase and hyaluronidase with IC_50_ values of 97.5 and 23.7 μg/mL, respectively [28]. These results suggest the potential of *S. muticum* ethyl acetate fraction to maintain skin structure and function.

#### 2.3.2. *Sargassum vachellianum*

The properties of moisture absorption and retention were tested in a test-tube experiment using the polyphenol-rich extract and polysaccharide-rich extract from *S. vachellianum* [70]. The effect of the polysaccharide-rich extract is stronger than polyphenol-rich extract on moisture absorption, but both of them time-dependently increased the weight of relative humidity [70]. They also time-dependently decreased the percentage of water loss compared to glycerol [70], indicating that *S. vachellianum* contributes to moisture absorption and retention abilities.

#### 2.3.3. *Sargassum horneri*

Several studies have reported that *S. horneri* extracts have the properties of moisturization and skin barrier repair [19,71,72]. A study has shown the skin hydration effects of an ethanol extract from *S. horneri* in fine dust-stimulated HaCaT keratinocytes [19]. Ethanol extract pretreatment increased the expression levels of proteins associated with skin moisture; involucrin, filaggrin, and lymphoepithelial kazal-type-related inhibitor [19]. Ethanol extract also reduced the protein levels of genes related to cutaneous inflammation and acidification of the stratum corneum, including protease-activated receptor-2, kallikrein-related peptidases, and phospholipase A2 in fine dust-induced cells [19]. Furthermore, the levels of tight junction proteins, including occludin, claudin-1, claudin-4, claudin-7, claudin-23, and zonula occludens-1, are increased with the treatment of ethanol extract [19]. These increments indicated the positive efficacy of ethanol extract in fine dust-impaired skin barrier function. Ethanol extract also exhibited hyaluronic acid increment, which prevents skin dryness by retaining water in the dermis [19].

Fucoidan is one of the functional compounds in *S. horneri*. There is a study showing the positive effects of fucoidan from *S. horneri* [71]. The skin moisture-controlling proteins are dose-dependently increased, and the protein levels related to cutaneous inflammation and acidification are decreased by the fucoidan fraction isolated from *S. horneri* [71]. Additionally, treatment with the fucoidan fraction increased the protein expression levels of tight junction proteins and hyaluronic acid [71]. The other in vitro study confirmed the effects of *S. horneri*-derived polysaccharide on moisture-preservation [72]. The polysaccharides from *S. horneri* have strong moisture-absorption and moisture-retention abilities as compared to propanediol and glycerin [72].

#### 2.3.4. *Sargassum confusum*

It has recently been confirmed that the fucoidan fraction of *S. confusum* suppressed the impairment of the skin barrier and stratum corneum hydration in UVB-stimulated keratinocytes [29]. These effects were associated with the recovery of skin barrier proteins in UVB-induced keratinocytes. Specifically, the treatment of the fucoidan fraction dose-dependently increased the expression levels of proteins related to skin moisture, including lymphoepithelial kazal-type-related inhibitor, involucrin, and filaggrin, and decreased the expression levels of kallikrein-related peptidase 5, protease-activated receptor-2, and phospholipase A2 [29]. Therefore, the fucoidan fraction of *S. confusum* can reduce skin barrier dysfunction and moisturization defects.

#### 2.3.5. *Sargassum glaucescens*

A study examined the moisture-retention and protective effects of *S. glaucescens* extracts in human primary epidermal keratinocytes [69]. *S. glaucescens* extracts treatment upregulated the expression levels of moisturizing-related genes such as transglutaminase 1, keratin 10, and keratin 14 in keratinocytes [69]. In addition, treatment with *S. glaucescens* extracts elevated natural moisturizing factor production in keratinocytes [69]. These effects are ascribed to the upregulation of filaggrin associated with the moisturization and barrier functions of the skin [74].

#### 2.3.6. *Sargassum fusiforme*

A study investigated the protective effects of *S. fusiforme* polysaccharides against UVB radiation-induced skin damage in hairless Kun Ming mice [23]. The data showed that *S. fusiforme* polysaccharides treatment significantly increased skin water content, spleen index, and thymus index in UVB-irradiated hairless Kun Ming mice [23]. The spleen and thymus are critical immune organs, and their indexes may reflect immunological functions [23]. Therefore, *S. fusiforme* polysaccharides have the potential to relieve UVB-induced skin moisture loss and maintain the immune homeostasis. *S. fusiforme* polysaccharides may also alleviate UVB-induced photoaging by reducing the thickness of the epidermis and dermis, attenuating the sebaceous hyperplasia, and disorganizing dermal collagen fiber symptoms [23].

### 2.4. Anti-Melanogenesis Activity

Melanin protects the skin from UV-induced skin damage. However, excessive accumulation of melanin can induce hyperpigmentation such as freckles, moles, and lentigo, and even lead to malignant melanoma [75]. Therefore, the discovery of anti-melanogenesis agents is needed to treat pigmentation and pigmentation disorders. Previous studies reported the potential anti-melanogenesis ability of *Sargassum* spp. extract (Table 5).

#### 2.4.1. *Sargassum polycystum*

A study tested the effects of *S. polycystum* on melanogenesis using α-melanocyte stimulating hormone (α-MSH)-stimulated a B16F10 murine melanoma cell line [41]. *S. polycystum* ethanol extract and hexane fraction inhibited cellular tyrosinase activity [41]. Additionally, hexane fraction reduced melanogenesis in B16F10 cells in both basal and α-MSH-stimulated conditions [41]. An in vivo study using guinea pigs showed that treatment with a cream containing ethanol extract and hexane fraction ameliorated UVB-induced hyperpigmentation [76]. Although further studies are necessary to reveal the molecular mechanisms, it appears that both ethanol extract and hexane fraction can be applied to anti-melanogenic agents.

#### 2.4.2. *Sargassum serratifolium*

A study revealed the hypopigmentation properties of ethanolic extract of *S. serratifolium* in B16F10 cells [81]. Researchers identified three anti-melanogenesis compounds including sargahydroquinoic acid, sargaquinoic acid, and sargacromenol in *S. serratifolium* ethanolic extract [81], and subsequent studies revealed that sargahydroquinoic acid and sargaquinoic acid are potent anti-melanogenesis components of *S. serratifolium* ethanolic extract [77,78].

The researchers further found the anti-melanogenesis mechanism of sargahydroquinoic and sargaquinoic acids in α-MSH-stimulated B16F10 cells [77,78]. Both compounds decreased tyrosinase activity, melanin content, and expression of melanogenic enzymes such as tyrosinase, tyrosinase-related protein-1 (TRP-1), and TRP-2 and attenuated the expression and phosphorylation of nuclear microphthalmia-associated transcription factor (MITF), a crucial transcriptional regulator of melanogenic enzymes [77,78]. In addition, both compounds inhibited the phosphorylation of nuclear cyclic adenosine monophosphate (cAMP) response element-binding protein, a transcriptional regulator of MITF expression, and decreased the production of intracellular cAMP, which plays a key role in PKA-dependent cAMP response element-binding protein activation in melanogenesis [77,78]. These results suggest that the anti-melanogenic activity of sargahydroquinoic acid and sargaquinoic acid may be achieved bycAMP-mediated downregulation of MITF [77,78].

#### 2.4.3. *Sargassum thunbergii*

A study examined the anti-melanogenesis effects of the *S. thunbergii* [36]. Ethanolic extract of *S. thunbergii* inhibited tyrosinase activity by 88.3% [36]. *S. thunbergii* ethanolic extract also significantly reduced the mRNA expression level of TRP-1 in B16F10 cells [36]. Although further mechanistic studies are necessary, *S. thunbergii* appears to have anti-melanogenic activity.

#### 2.4.4. *Sargassum fusiforme*

Several studies have confirmed that *S. fusiforme* extracts and derived compounds exhibit anti-melanogenesis effects [24]. A study investigated the anti-melanogenesis effects of sulfated polysaccharides isolated from Celluclast-assisted extract of *S. fusiforme* in α-MSH-induced B16F10 melanoma cells [24]. Sulfated polysaccharides decreased α-MSH-stimulated melanin contents and intracellular tyrosinase activity in a dose-dependent manner [24]. In addition, sulfated polysaccharides inhibited the expression of melanogenesis-related proteins such as MITF, tyrosinase, TRP-1, and TRP-2 in α-MSH-induced B16F10 cells [24,79,80].

Another study evaluated the melanogenesis inhibitory effect of fucoidan isolated from *S. fusiforme* in α-MSH-stimulated B16F10 melanoma cells [79]. The data indicated that fucoidan significantly inhibited α-MSH-mediated intracellular melanin content by reducing the level of tyrosinase activity [79]. The anti-melanogenic mechanism of fucoidan is associated with the inhibition of tyrosinase, TRP-1, and TRP-2 expression by activating the ERK-MAPK signaling pathway and thus inhibiting nuclear translocation of MITF [79].

Other studies investigated the anti-melanogenesis effects of MeOH fraction isolated from *S. fusiforme* in a three-dimensional human skin model [80]. The MeOH fraction showed potent mushroom tyrosinase inhibitory activity with an IC_50_ value of 1.3 µg/mL [80].The treatment of the MeOH fraction (5–20 mg/mL) dose-dependently inhibited the skin pigmentation in the three-dimensional human skin model without cytotoxic effects [80]. Based this, *S. fusiforme* may be applied to cosmeceutical products.

#### 2.4.5. *Sargassum siliquastrum*

A study investigated the melanogenesis inhibitory effect of aqueous extracts of *S. siliquastrum* in B16F10 melanocytes and zebrafish [32]. According to the results, *S. siliquastrum* aqueous extract showed relatively strong mushroom tyrosinase inhibitory activity with an IC_50_ value of 19.85 μg/mL [32]. Furthermore, *S. siliquastrum* aqueous extract decreased tyrosinase activity and melanin synthesis in B16F10 cells and zebrafish embryos [32]. Therefore, these results showed that the *S. siliquastrum* aqueous extract could be utilized as a natural source of skin-whitening agents.

## 3. Conclusions

The studies covered in this review suggest that *Sargassum* spp. extract or derived compounds have the potential to prevent and improve skin aging by elevating antioxidant, photoprotective, anti-inflammatory, anti-melanogenesis, moisturizing, and skin barrier repair properties. Although further studies including clinical trials should be performed to reveal more detailed mechanisms and safe dosages underlying *Sargassum* spp.-mediated anti-skin aging effects, they may be applied to cosmeceutical and pharmaceutical products.

## Figures and Tables

**Table 1:** Bioactive components and biological properties of major *Sargassum* spp.

Species	Bioactive Compounds	Biological Properties	References
*S. horneri*	phlorotannins polysaccharide (alginates) plastoquinone (sargachromenol) monogalactosyldiacyl-glycerols proteoglycans	antioxidant anti-photoaging, anti-inflammatory anti-obesity, anti-atopic dermatitis skin barrier repair	[15,16,17,18,19]
*S. fusiforme*	plastoquinones polysaccharide (alginates) carotenoid (fucoxanthin) lectin glycyrrhizin fucosterol saringosterol	antioxidant anti-photoaging, anti-inflammatory anti-obesity, antidiabetic activity anti-melanogenesis, skin barrier repair	[20,21,22,23,24]
*S. muticum*	phlorotannins meroterpenoids polysaccharides (fucoidans, alginate, laminarin)	anti-winkle anti-obesity, anti-atopic dermatitis skin barrier repair	[25,26,27,28]
*S. pallidum* *(S. confusum)*	polysaccharides (alginates, fucoidan) oligosaccharides	anti-photoaging antidiabetic activity, skin barrier repair	[29,30]
*S. siliquastrum*	chromanols (sargachromanol D, E, K) Sulfated polysaccharide (fucoidan) carotenoid (fucoxanthin)	antioxidant anti-photoaging, anti-melanogenesis	[31,32]
*S. thunbergii*	phlorotannins polysaccharides sulfated galactofucan isopentadiene	antioxidant anti-obesity, anti-cancer anti-melanogenesis	[33,34,35,36]
*S. fulvellum*	phlorotannins (fucols, phlorethols, fucophlorethols), polysaccharides (fucoidan, alginates, laminaran), carotenoid (fucoxanthin)	antioxidant anti-inflammatory	[37,38]
*S. polycystum*	polysaccharides (fucoidan, alginates)	antioxidant antidiabetic activity, anti-melanogenesis anti-cancer, antibacterial	[39,40,41,42,43]
*E. stolonifera*	phlorotannin (dieckol, eckol, phlorofucofuroeckol A, B)	antioxidant anti-inflammatory anti-obesity anti-cancer	[44,45,46]

**Table 2:** Antioxidant and photoprotective activities of *Sargassum* spp.

Type of *Sargassum*	Solvent	Model	Conc.	Effects	Active Component	Ref.
*S. muticum*	80% EtOH	Male HR-1 strain hairless mice	100 mg/kg body weight	↓ average length and depth of wrinkles ↓ epidermal thickness ↑ collagen bundle formation	ND	[25]
		HaCaT cells	ND	↓ collagen degradation		
*S. cristafolium*	EtOH	FemaleBALBL/c mice	20 μM	↓ wrinkles and desquamation ↑ skin healing process ↑ antibacterial activity	fucoxanthin	[47]
*S. siliquastrum*	chloroform and MeOH 1:1	HaCaT cells	25–100 μg/mL	↓ oxidative damage ↓ apoptosis	fucoidan	[31]
*S. fusiforme*	80% EtOH	HaCaT cells	31.25–125 μg/mL	↓ oxidative damage ↓ inflammatory responses ↓ collagen degradation	polysaccharides	[48]
	EtOH	HDF cells	25–100 μg/mL	↓ oxidative damage ↑ cell viability ↑ collagen synthesis	sulfated polysaccharides	[20]
*S. confusum*	chloroform and MeOH 1:1	HaCaT cells	25–100 μg/mL	↓ oxidative damage ↑ cell viability ↓ stratum corneum hydration damage	fucoidan	[29]
*S. coreanum*	EtOH	HaCaT cells	25–100 μg/mL	↓ oxidative damage ↓ apoptosis	fucoidan	[49]
*S. horneri*	80% MeOH	HaCaT cells	31.6–125 μg/mL	↓ oxidative damage ↑ cell viability ↓ apoptosis	HTT, apo-9′-fucoxanthinone	[15]
	70% EtOH	HDF cells	50–200 μM	↓ oxidative damage ↓ apoptosis ↓ inflammatory responses ↓ degradation of ECM components	HTT	[50]
	80% MeOH	HaCaT cells	6.25–25 μg/mL	↓ oxidative damage ↑ cell viability ↓ apoptosis	(-)-loliode	[51]
		HDF cells		↓ oxidative damage ↑ collagen synthesis		
		zebrafish		↓ oxidative damage		
	chloroform and MeOH 1:1	HaCaT cells	25–100 μg/mL	↓ oxidative damage ↑ cell viability ↓ apoptosis	fucoidan	[52]
	ND	HDF cells	5–20 μM	↓ skin cell damage ↓ collagen degradation	sargachromenol	[53]
	MeOH	HDF cells	5–20 μM	↓ oxidative damage ↓ collagen degradation	sargachromanol E	[54]

MeOH, methanol; HTT, 6-hydroxy-4,4,7a-trimethyl-5,6,7,7a-tetrahydrobenzofuran-2(4H)-one; EtOH, ethanol; HDF, human dermal fibroblast; ECM, extracellular matrix; ND, not determined; HR-1, hairless-1; ↑, increase; ↓, decrease.

**Table 3:** Anti-inflammatory activities of *Sargassum* spp.

Type of *Sargassum*	Solvent	Model	Conc.	Effects	Active Component	Ref.
*S. fulvellum*	95% EtOH	HaCaT cells	30–100 μg/mL	↑ antioxidant defenses ↓ inflammatory responses	ethyl acetate fraction	[38]
		BALB/c mice	3–10 μg			
	96% EtOH	RAW264.7 cells	10–50 μg/mL	↓ inflammatory responses	hexane fraction	[61]
		ICR mice	45 μg/ear	↓ mouse ears edema		
*S. horneri*	70% EtOH	HaCaT cells	25–100 μg/mL	↓ inflammatory responses ↓ oxidative damage	alginic acid	[16]
*M. myagroides*	96% EtOH	RAW264.7 cells	10–100 μg/mL	↓ inflammatory responses	6,6′-bieckol	[62]
		ICR mice	90 μg/ear	↓ mouse ears edema		
	96% EtOH	RAW264.7 cells	25–100 μg/mL	↓ inflammatory responses	phlorofucofuroeckol B	[63]
		ICR mice	90 μg/ear	↓ mouse ears edema		
	96% EtOH	RAW264.7 cells	0.3–1.2 μM	↓ inflammatory responses	sargaquinoic acid	[64]
*E. stolonifera*	96% EtOH	RAW264.7 cells	50–200 μg/mL	↓ inflammatory responses	phlorofucofuroeckol A and B	[44]
*S. macrocarpum*	80% EtOH	RAW264.7 cells	0.4–0.8 μM	↓ pro-inflammatory activity ↓ oxidative damage	sargahydroquinoic acid	[65]
		mice	10–15 mg/mL	↓ pro-inflammatory activity		
*S. fusiforme*	EtOH	RAW264.7 cells	25–100 μg/mL	↓ inflammatory responses	sulfated polysaccharides	[24]
*S. micracanthum*	80% EtOH	RAW264.7 cells	12.5–100 μM	↓ inflammatory responses	sargachromenol	[66]
*S. binderi*	95% EtOH	RAW264.7 cells	25–100 μg/mL	↓ cell death ↓ oxidative stress ↓ inflammatory activity	sulfated polysaccharides	[67]
		zebrafish larvae				

EtOH, ethanol; ICR, institute of cancer research; ↑, increase; ↓, decrease.

**Table 4:** Moisturization and Skin barrier repair activities of *Sargassum* spp.

Type of *Sargassum*	Solvent	Model	Conc.	Effects	Active Component	Ref.
*S. muticum*	EtOH:water (70:30)	enzyme activity test	200 μg/mL	↓ collagenase activity ↓ hyaluronidase activity	ethyl acetate fraction	[28]
*S. vachellianum*	90% EtOH	enzyme activity test	200–1000 μg/mL	↑ moisture absorption ↑ moisture retention rate	polyphenol, polysaccharide	[70]
*S. horneri*	70% EtOH	HaCaT cells	31.3–125 μg/mL	↑ skin moisture ↓ cutaneous inflammation ↓ acidification of stratum corneum ↓ skin dryness	polyphenol	[19]
	95% EtOH		12.5–50 μg/mL		fucoidan	[71]
	60% EtOH	ND	ND	↑ moisture absorption ↑ moisture retention rate	polysaccharide	[72]
*S. confusum*	Chloroform:MeOH (1:1)	HaCaT cells	25–100 μg/mL	↓ skin barrier dysfunction ↓ moisturization defects	fucoidan	[29]
*S. glaucescens*	water	human primary epidermal keratinocytes	0.03125 mg/mL	↑ retention of moisture	ND	[69]
*S. fusiforme*	95% EtOH	hairless Kun Ming mice	200–600 mg/kg	↓ skin moisture loss ↑ immunological function ↓ thickness of epidermis and dermis ↓ sebaceous hyperplasia ↓ dermal collagen fibers symptoms	polysaccharides	[23]

EtOH, ethanol; ND, not determined, MeOH, methanol; ↑, increase; ↓, decrease.

**Table 5:** Anti-melanogenesis activities of *Sargassum* spp.

Type of *Sargassum*	Solvent	Model	Conc.	Effects	Active Component	Ref.
*S. polycystum*	95% EtOH	B16F10 cells	100–500 µg/mL	↓ intracellular tyrosinase activity ↓ melanin contents	hexane fraction	[41]
		Guinea pig	130 µg/mL	↓ pigmentation		[76]
			72 µg/mL	↓ pigmentation	ND	
*S. serratifolium*	70% EtOH	B16F10 cells	1–4 µM	↓ intracellular tyrosinase activity ↓ melanin contents	sargahydroquinoic acid, sargaquinoic acid	[77,78]
*S. thunbergii*	53.5% EtOH	B16F10 cells	1–2 mg/mL	↑ mushroom tyrosinase inhibition activity ↓ melanogenic activity	ND	[36]
*S. fusiforme*	EtOH	B16F10 cells	12.5–50 µg/mL	↓ intracellular tyrosinase activity ↓ melanin contents	sulfated polysaccharide	[24]
			25–100 µg/mL	↑ mushroom tyrosinase inhibition activity ↓ melanin contents	fucoidan	[79]
	hexane and dichloromethane 1:1	3D human skin models	5–20 mg/mL	↑ mushroom tyrosinase inhibition activity ↓ pigmentation	MeOH fraction	[80]
*S. siliquastrum*	Water	B16F10 cells	100 µg/mL	↑ mushroom tyrosinase inhibition activity ↓ intracellular tyrosinase activity ↓ melanin contents	ND	[32]
		Zebrafish embryo				

EtOH, ethanol; ND, not determined; 3D, three-dimensional; ↑, increase; ↓, decrease.

## Data Availability

The datasets generated for this study are available on request to the corresponding author.
